# Husbands' Plan to Participate in Birth Preparedness and Complication Readiness in Haramaya Health and Demographic Surveillance System Site, Eastern Ethiopia: A Community-Based Cross-Sectional Study

**DOI:** 10.3389/fpubh.2022.856809

**Published:** 2022-04-18

**Authors:** Seada Sufian, Mohammed Abdurke Kure, Merga Dheresa, Adera Debella, Bikila Balis, Kedir Teji Roba

**Affiliations:** ^1^Department of Nursing, Harar Health Science College, Harar, Ethiopia; ^2^School of Nursing and Midwifery, College of Health and Medical Sciences, Haramaya University, Harar, Ethiopia

**Keywords:** husband participation, birth preparedness, complication readiness, associated factor, Ethiopia

## Abstract

**Background:**

Partner involvement in maternal health services utilization remains a major public challenge in the developing world. Strategies of involving men in maternal health services are a critical and proven intervention for reducing maternal and neonatal mortality by ensuring safe delivery and reducing complications during childbirth. Moreover, the husbands' involvement during pregnancy helps their spouses to make timely decisions and avoid maternal delays, especially first and second delays. Although birth and complication readiness have been studied in developing countries such as Ethiopia, almost all previous researchers were focused primarily on women participants. Therefore, we aimed to investigate factors associated with husband involvement in birth preparedness and complication readiness plan in Haramaya Health and Demographic Surveillance site, Eastern Ethiopia.

**Methods:**

A community-based cross-sectional study was conducted from March 1 to 30, 2020 among men whose wives were pregnant in Haramaya Health and Demographic Surveillance (HDSS) site in Eastern Ethiopia. The calculated sample size was 653, however while contacting 653 husbands only 630 had given the full interview, hence 630 respondents were remained in the analysis. Participants were approached through a systematic sampling technique. Data were collected using a pre-tested structured questionnaire through a face-to-face interview, and entered into Epidata version 3.1 and analyzed using SPSS version 22 (IBM SPSS Statistics, 2013). The prevalence was reported using proportion with 95% Confidence Interval (CI) and summary measures. Predictors were assessed using a multivariable logistic regression analysis model and reported using an adjusted odds ratio (AOR) with 95%CI. Statistical significance was declared at *p* < 0.05.

**Results:**

Overall, the prevalence of the husband's plan to participate in birth preparedness and complication readiness was 59.6% (95%CI:56–64%). In the final model of multivariable analysis, predictors like husband's knowledge of birth preparedness and complication readiness [AOR = 4.18, 95%CI:2.05, 8.51], having a discussion with spouse on the place of delivery [AOR = 6.84, 95% CI: 4.17, 11.22], husband's knowledge of danger signs during labor and delivery [AOR = 3.19, 95 % CI: 1.52, 6.71], and making a postpartum plan[AOR = 2.30, 95 % CI: 1.38, 3.85] were factors statistically associated with husband's plan to participate in birth preparedness.

**Conclusions:**

This study pointed out that two in every five husbands failed to plan birth preparedness and complication readiness. As a result, all stakeholders should emphasize male partners' education in terms of birth preparedness and complication readiness, as well as knowledge of danger signs during labor and delivery. They should also encourage male partners to discuss a place of delivery and have a postpartum plan in place to reduce potential complications related to labor and delivery.

## Introduction

Globally, approximately 287,000 mothers die each year as a result of pregnancy and childbirth complications. The vast majority of these maternal deaths (99%) occur in developing countries, with Sub-Saharan Africa alone accounting for 66% (1). These deaths are the result of complications during pregnancy, childbirth, or postpartum. Studies have shown that making a birth plan that includes birth-preparedness and complication readiness measures for pregnant women, their spouses, and their families are found to be a key strategy that can reduce the number of women dying from such complications (1, 2).

Birth preparedness and complication readiness (BPCR) is a comprehensive package aimed at promoting timely access to skilled maternal and neonatal services, as well as encouraging pregnant women and their families to actively prepare for and make decisions about delivery (2). This stems from the fact that every pregnant woman is at risk of unexpected and life-threatening complications that could result in death or injury to herself or her infant (3). According to various research reports, pregnancy and childbirth are still regarded solely as women's issues in Sub-Saharan Africa because husbands have tremendous control over the women's decision process both socially and economically (4–7). On the contrary, evidence has shown that involving men in maternal health care has positive outcomes such as reduced maternal morbidity and mortality due to a sufficient birth plan, thereby avoiding care-seeking delays due to obstetric emergencies (8–11), increased institutional deliveries (5, 9, 10) and postnatal service utilization (9, 12). With these all interventions, three phases of maternal delay can be averted.

Literature has shown that numerous factors affect the husband's involvement in the process of BPCR planning during pregnancy and childbirth. For instance, previous research report identified local factors (societal perception such as considering childbirth as a natural process, pregnancy and childbirth are women's role, preference for traditional birth attendance's care and novelty of the idea of husband's involvement in pregnancy and birth care) as barriers of husband's plan to participate in BPCR (13–15). In Sub-Saharan Africa like Ethiopia, despite its public health importance for birth outcomes, male participation in maternal and child health (MCH) remains low, and having a male partner (MP) present in the labor room during delivery is utterly impossible in many settings (9, 11, 16), even in a limited area like urban setting, where male partners have been supportive to their spouses, there are unwelcoming, intimidating, and unsupportive health systems, presenting a missed opportunity, which embarrasses their commitment (13, 17). Similarly, researchers have reported that factors such as poor timely action by family, which leads to a great deal such as looking for a source of money and potential blood donors in case of emergency, finding for transportation, and reaching the appropriate referral facility remain a major challenge in Africa (14, 18).

In Ethiopia, husbands have strong decision-making power over their spouses both at the community and at the household level, and traditionally women have little independence in decisions making process. Cultural barriers, knowledge of recognizing potential complications, and facility service factors all contribute to the husband's involvement in birth preparation and complication readiness planning (19). According to previous studies conducted in limited areas of Ethiopian regions, husband disapproval for antenatal care accounts for 15.5% of the factors influencing antenatal care, and only 21% of pregnant women were accompanied by their husbands to visit the ANC clinic (13). As a result, male participation in birth preparation and complication preparation will be critical in reducing maternal mortality (20). Male involvement allows men to encourage their wives to use obstetric services, and the couple will be better prepared for birth complications. This would result in a reduction in all three phases of delay: delay in deciding to seek care, delay in getting to care, and finally delay in receiving care. In developing countries, the male partner can play an important role, particularly in the first and second stages of delay, and thus positively impact birth outcomes (21–23).

In Ethiopia, in the last decade, various studies have been conducted to assess the practice of birth preparation and complication readiness among mothers; with little attention have been given to the level of the husband's involvement in birth preparation. Moreover, although numerous factors associated with male involvement in BPCR have been identified in the various research report (13, 14, 24, 25), in Ethiopia, husbands' participation in the BPCR received little policy attention (23). In addition, the majority of the previous studies were facility-based and primarily focused on emergency obstetric care and other routine services (26). Therefore, this study was aimed to investigate husbands' participation in BPCR and its associated factors among husbands whose wives were pregnant in the Haramaya HDSS site, Eastern Ethiopia.

## Methods and Materials

### Study Design, Setting, and Period

A community-based cross-sectional study was conducted from March 1 to March 30, 2020, in four kebeles (the smallest administrative unit) in Ethiopia, namely Biftu-Geda, Ifa-Oromia, Gobe-Chala, and Kuro found in Haramaya districts, which were located 500 km away from Addis Ababa, capital city Addis Ababa. The 2007 national census reported the total population for this District is 271,018, of whom 138,282 were men and 132,736 were women (27). The Haramaya Health and Demographic Surveillance Site which is maintained by Haramaya University was established in the year 2018 GC. The site was established on 12 rural kebeles of Haramaya district. The site constitutes 93,363 residents and 1,712 pregnant women (28).

### Study Participants

All husbands whose wives were pregnant in the Haramaya HDSS site during the study period were considered as source population. The study population consisted of all systematically selected husbands whose wives were in their third trimester of pregnancy in the Haramaya district HDSS site's selected kebeles. Husbands who were not staying with their wives during pregnancy and childbirth and, those who were critically ill and unable to provide the required information during data collection were excluded from the study.

### Sample Size and Sampling Procedure

In this study, the maximum required sample size was calculated using the single population proportion formula by considering the following assumptions. Taking the prevalence of husband involvement in BPCR (45%, P=0.45) from previous a study conducted in Wolaita Sodo town, Southern Ethiopia (23), a 95% confidence level (Z_α/2_ =1.96), to increase the representativeness of the sample size and to boost the precision, 4%(α = 0.04) tolerable margin of error was considered. Thus,


$$\begin{array}{l} n=\frac{za/2^{2}\;p\left(1-p\right)}{d^{2}}=\frac{{\left(1.96\right)}^{2}\left(0.45\right)\left(1-0.45\right)}{0.04^{2}}=594 \end{array}$$

By adding a 10% contingency for the non-response rate, the calculated sample size was 653, however while contacting 653 husbands only 630 had given the full interview, hence 630 respondents were remained in the analysis.

**Figure 1:**
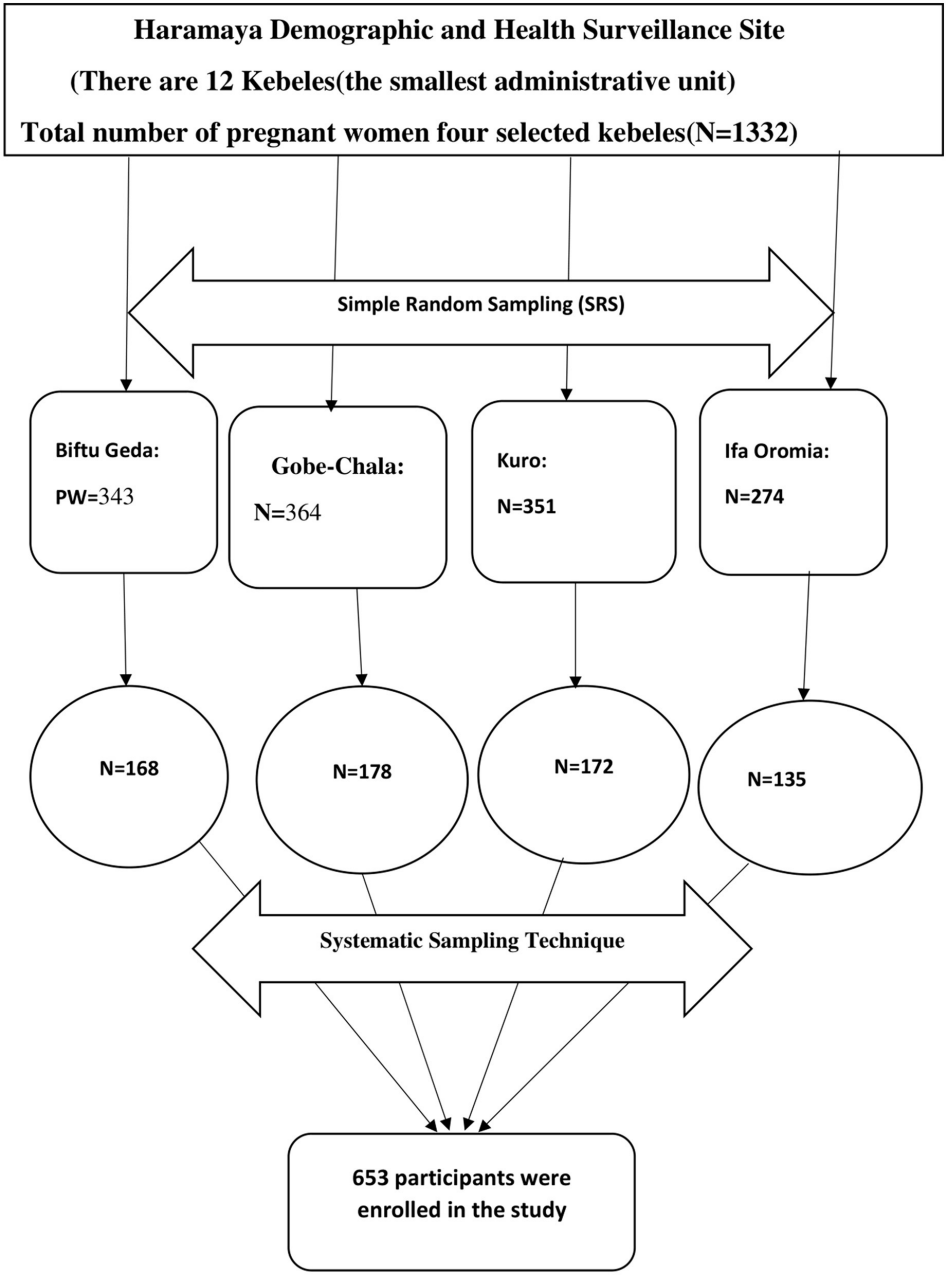
The schematic diagram of the sampling procedure for the study on husbands' plan to participate in birth preparedness and Complication Readiness in Haramaya Health and Demographic Surveillance System Site, Eastern Ethiopia.

In this study, two-stage sampling was used. Initially, a simple random sampling technique was used to select four kebeles from a total of 12 rural kebeles of the Haramaya HDSS site. To do so, the Haramaya HDSS database maintained by Haramaya University was used as a sampling frame to identify a list of pregnant women in each kebele. The total number of pregnant women who are living with their spouse in these four kebeles is 1,332, of which Biftu-Geda, Gobe-Chala, Kuro, and Ifa Oromia have 343, 364, 351, and 274 pregnant women respectively (28). Then, the house of pregnant women was traced to identify the study participants (Households with the husbands of pregnant women). The calculated sample size was proportionally allocated to the four selected kebeles. The systematic sampling technique was employed to select the households, and the first house was selected using lottery methods. For absent participants, rescheduling was done to conduct the interview again. If the selected household does not fulfill the inclusion criteria, the next household was substituted for our study and if more than one candidate was available in the single household, one of them was interviewed by lottery method. Accordingly, a total of 653 sample sizes was proportionally allocated to each kebele to obtain the required numbers of an individual to be included in the estimated sample from each aforementioned kebeles. Then, the K^th^ interval was calculated for all selected kebeles (K^th^ = 1,332/653 = ≈2). The sequence of the “k^th^” interval was 2 for all selected kebeles. Therefore, every second eligible participant was interviewed, and data were collected until the required sample size was obtained (Figure 1).

### Data Collection Tool and Procedure

The data were collected through a face-to-face interview with a pretested structured questionnaire developed after a review of the literature (29). The questionnaires asked about socioeconomic and demographic information, knowledge of danger signs during pregnancy, labor and delivery, and postnatal care, knowledge of BPCR, and plans to participate in birth preparedness and complication readiness. Ten Bachelor of Science (BSc) nurses were collected the data after a five-day training on the tools and survey methods.

### Study Variables and Measurements

**Dependent variable:** In this study, the outcome variable was husbands' plan to participate in birth preparedness and complication readiness (Yes/No). The outcome variable was dichotomized as 1 and 0. Thus, it was recoded into binary outcomes as “good participation = 1” and “poor participation = 0”. **Independent variables:** In this study, the explanatory variables were categorized as: demographic and socioeconomic characteristics (age, religion, number of children and occupation, level of education, and marital status), **obstetric related factors**: Knowledge of husbands on key danger signs of pregnancy, labor and the postpartum period, knowledge of husbands on birth preparedness and complication readiness.

### Operational Definitions and Measurements

**Birth preparedness and complication readiness**: is a strategy to encourage husbands to know the signs of obstetric complications and emergencies, to choose a preferred birthplace and attendant at birth, to arrange for transportation to the skilled care site in the event of emergence, to save or arrange alternate funds for the cost of the emergence, and to accompany her to the emergence care. identifying a blood donor and preparing clean clothes for the mother and child (30). **Husbands' plan to participate in BPCR**: was measured by nine items, those who responded 'yes' scored 1 and if 'No' scored 0 then the respondents who were scored above the mean value of the indicators of BPCR (23). Good participation in BPCR: Those husbands who practiced five and above elements of nine items (23). Poor participation in BPCR: Husbands who practiced four or fewer elements of nine items (23).

**Knowledge of danger signs:** According to WHO, there are 10 danger signs of pregnancy, delivery, and postnatal period. **Better knowledge**: In this study, the respondents who knew greater than or equal to five danger signs during pregnancy, delivery, and postnatal period which is above the mean value (danger signs assigned from 1 to 10) were categorized as ‘have better knowledge” (31). **Good knowledge**: The respondent who knew below the mean value (<5 danger signs) were considered as “have a good knowledge” (31). **Poor knowledge**: In this study, the husband's poor knowledge about danger signs can be defined as if the respondent did not know any listed danger signs, they were considered as “poor knowledge” (31).

**Husband's knowledge of BPCR:** According to the WHO, there are 9 items to assess the components of BPCR. **Better knowledge**: In this study, if the husbands mentioned five and above components of BPCR items, which is the mean value (component assigned 1- 9), they categorized as “have better knowledge” (31)**. Good knowledge**: The respondents who knew below mean value (<5 components) of the nine BPCR items, they considered as “have good knowledge” (31). **Poor knowledge**: In this study, if the respondents did not know any components of Birth preparedness and complication readiness, we categorized them as “ have poor knowledge” (31).

### Data Quality Control

The questionnaire was initially prepared in English and then translated into the local languages by a bilingual expert (Afaan Oromoo language). Then, it was translated back into an English version to ensure its consistency. The data collectors and field supervisors received training on the data collection tool and procedures. Before the actual study data collection, the pretest was conducted among 3% of husbands whose wives were pregnant in similar settings. The investigators and experienced field research supervisors provided regular supervision.

### Data Processing and Analysis

First, the collected data were checked for completeness, consistency. Then, they were cleaned, coded, and entered into EpiData version 3.1 for further analysis. The entered data were exported to SPSS version 22 for analysis. Descriptive and summary statistics were conducted and reported using frequency tables and figures. The outcome variable was recoded into binary outcome as “good participation = 1” and “poor participation = 0”. A binary logistic regression model was fitted to check for an association between independent variables and the outcome variable. The model fitness was checked by Hosmer-Lemeshow statistics and Omnibus tests. A multivariable analysis was performed to identify the true predictors of the husband's plan to participate in the BPCR plan. A multi-collinearity test was carried out to check the presence of correlation between independent variables by using the standard error and co-linearity statistics, and no collinearity effects were detected. Thus, the value of the Variance Inflation Factor (VIF) was 0.951. The direction and strength statistical association was measured by odds ratio (OR) along with the 95% confidence interval (CI). A *p* < 0.05 was considered to declare statistical significance both in bi-variable and multivariable analysis.

## Results

### Socio-Demographic Characteristics of the Respondents

A total of 630 husbands were enrolled in this study, with a response rate of 96.5 %. The age of the study participants ranged from 18 to 60 years with a mean age of 31.7 (SD = ±7.4). The majority of the study participants, 387 (61.4%) were between the ages of 30 and 39 years. Four hundred-thirty (68.3%), two hundred-forty (38.1%), and five hundred-eight-four (92.7%) of the husbands were farmers, had no formal education, and were married in monogamous marriages, respectively. More than half of the participants, 356 (56.5 %) had 1–4 children in their family (Table 1).

**Table 1:** Socio-demographic characteristics of husbands whose wives were pregnant in Haramaya HDSS site, Eastern Ethiopia, 2020.

**Characteristics**	**Categories**	**Frequency(*n*)**	**Percentage (%)**
Husband's age (years)	18–29	38	6.0
	30–39	387	61.4
	40–49	177	28.2
	≥ 50	28	4.4
Religion	Muslim	611	97
	Orthodox	15	2.4
	Protestant	4	0.6
Number of children	Have no children	85	13.5
	1–4	356	56.5
	>4	189	30.0
Husband's educational level	No formal education	240	38.1
	Primary (1–8)	194	30.8
	Secondary (9–12)	92	14.6
	College and above	104	16.5
Husband's occupation	Farmers	430	68.3
	Merchant	97	15.4
	Government employee	69	11.0
	Other^*^	34	5.3
Marital status	Monogamous	584	92.7
	Polygamous	46	7.3
Wife's educational level	No formal education	344	54.6
	Primary (1–8)	160	25.4
	Secondary (9–12)	62	9.8
	College and above	64	10.2

* *Daily laborer, private employees*.

### Husband's Knowledge Status on Key Danger Signs During Pregnancy, Labor and Delivery, Postnatal Period

Regarding obstetric danger signs, more than half of the respondents, 352 (55.9%) mentioned five and more danger signs during delivery and around 195(31.0%) of them listed five and more danger signs during the postnatal period. Similarly, nearly half 272(43.2%) of the husbands had responded to five and more BPCR components. Five hundred ninety-seven (94.8%) of the participants had awareness of the significance of ANC during pregnancy (Table 2).

**Table 2:** Knowledge of obstetric danger signs related factors among husbands whose wives were pregnant in Haramaya HDSS site, Eastern Ethiopia, 2020.

**Characteristics**	**Categories**	**Frequency**	**Percentage**
		**(*n*)**	**(%)**
Husband's knowledge status on danger signs during pregnancy	Poor knowledge	58	9.2
	Good knowledge	248	39.4
	Better knowledge	324	51.4
Husband's knowledge status on danger signs during labor and delivery	Poor knowledge	79	12.5
	Good knowledge	199	31.6
	Better knowledge	352	55.9
Husband's knowledge status on danger signs during postnatal care	Poor knowledge	151	44.0
	Good knowledge	284	45.1
	Better knowledge	195	31.0
Husband's knowledge status on components of BPCR	Poor knowledge	130	20.6
	Good knowledge	228	36.2
	Better knowledge	272	43.2
Husband's knowledge on importance of ANC follow-up	Yes	597	94.8
	No	33	5.2

### Husband's Source of Information About Birth Preparedness and Complication Readiness Plan

**Figure 2:**
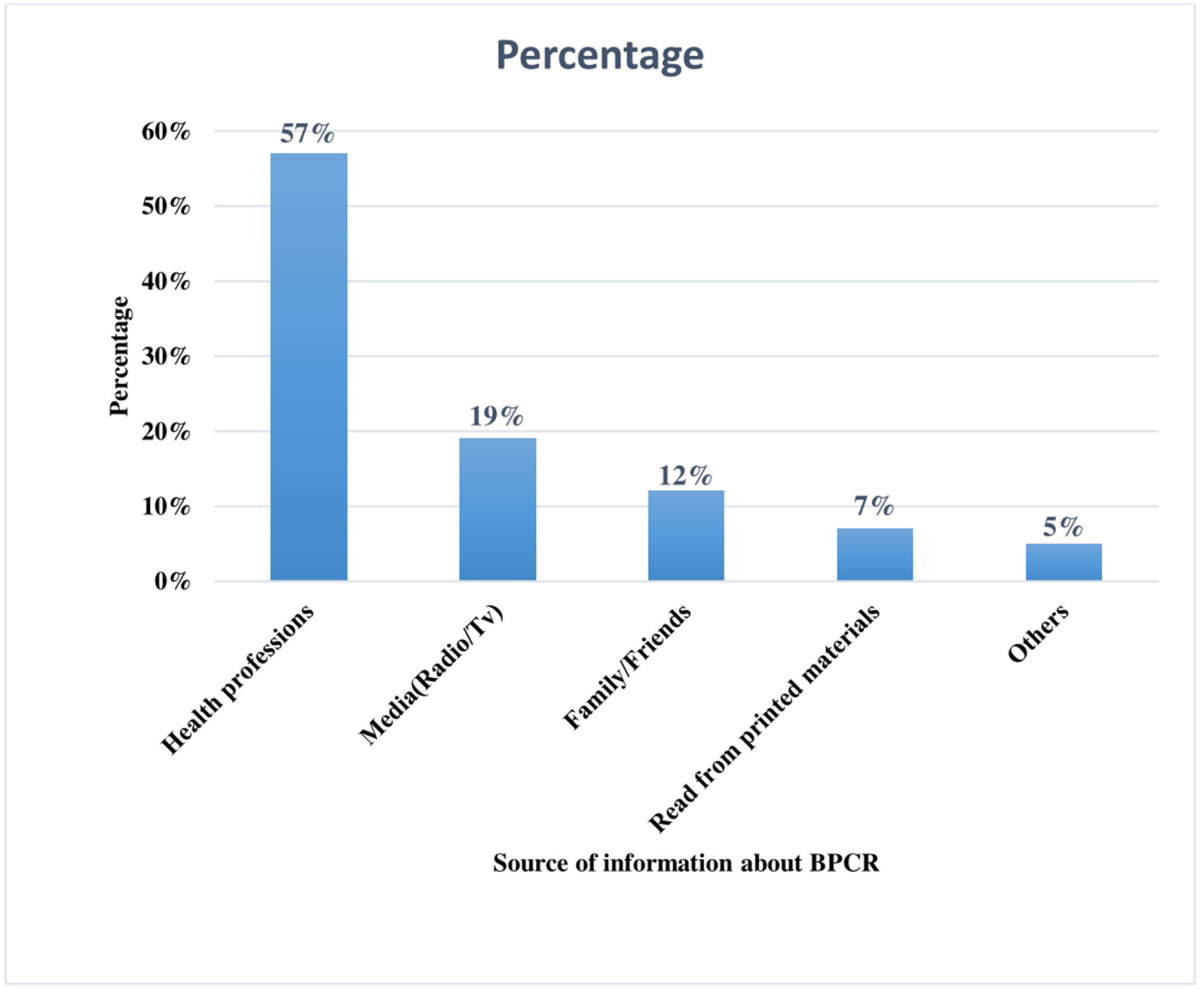
Source of information about birth preparedness and complication readiness Haramaya Health and Demographic Surveillance System site, Eastern Ethiopia, 2020.

In this study, the respondents were also assessed for their source of information about birth preparation and complication readiness. Accordingly, of the total 630 study participants enrolled in this study, more than half (57.16%) of them were heard from health care providers followed by media (Radio/TV) (18.53%), family/friends (12.41%), reading printed materials (7.17%), and the remaining 4.73% of the heard from other sources in their life (Figure 2).

### Husband's Plan to Participate in Birth Preparedness and Complication Readiness Plan

In this study, the husband's status of birth preparation and complication readiness plan during labor, delivery, and postpartum were assessed. Accordingly, of the 630 husbands who participated in the study, nearly half, 334(53.0%) of the respondents were made a plan for the place of delivery, and three hundred-four (48.3%) of them identified skilled birth attendants during labor and delivery. Nearly two-thirds (64.9%) of the respondents stated that they save money for delivery. Almost one-third (33.0%) of husbands had a plan for blood donation and 302 (47.9%) had a plan to accompany their wives during labor and delivery. Likewise, two hundred sixty-seven (42.4%) of the husbands made a transportation plan, and nearly half (49.7%) of them made a postpartum plan during their current pregnancy (Table 3).

**Table 3:** Husband's plan to participate in birth preparedness and complication readiness plan in Haramaya HDSS site, Eastern Ethiopia, 2020.

**Characteristics**	**Category**	**Frequency (*n*)**	**Percentage (%)**
Identify a place of delivery	Yes	334	53.02
	No	296	46.98
Discussed with spouse on place of delivery	Yes	360	57.7
	No	270	41.3
Identify skilled birth attendants at delivery	Yes	304	48.25
	No	326	51.75
Save money for delivery	Yes	409	64.93
	No	221	35.07
Identify potential blood donors	Yes	209	33.17
	No	421	66.83
Identify birth accompany for delivery	Yes	302	47.93
	No	328	52.07
Prepare mode of transportation during labor	Yes	267	42.38
	No	368	57.62
Make a postpartum plan following delivery	Yes	318	50.47
	No	312	49.53
Save money for emergency during labor and delivery	Yes	231	36.67
	No	399	63.33
Identify need of ANC during pregnancy	Yes	597	94.7
	No	33	5.3

*ANC, Antenatal Care*.

**Figure 3:**
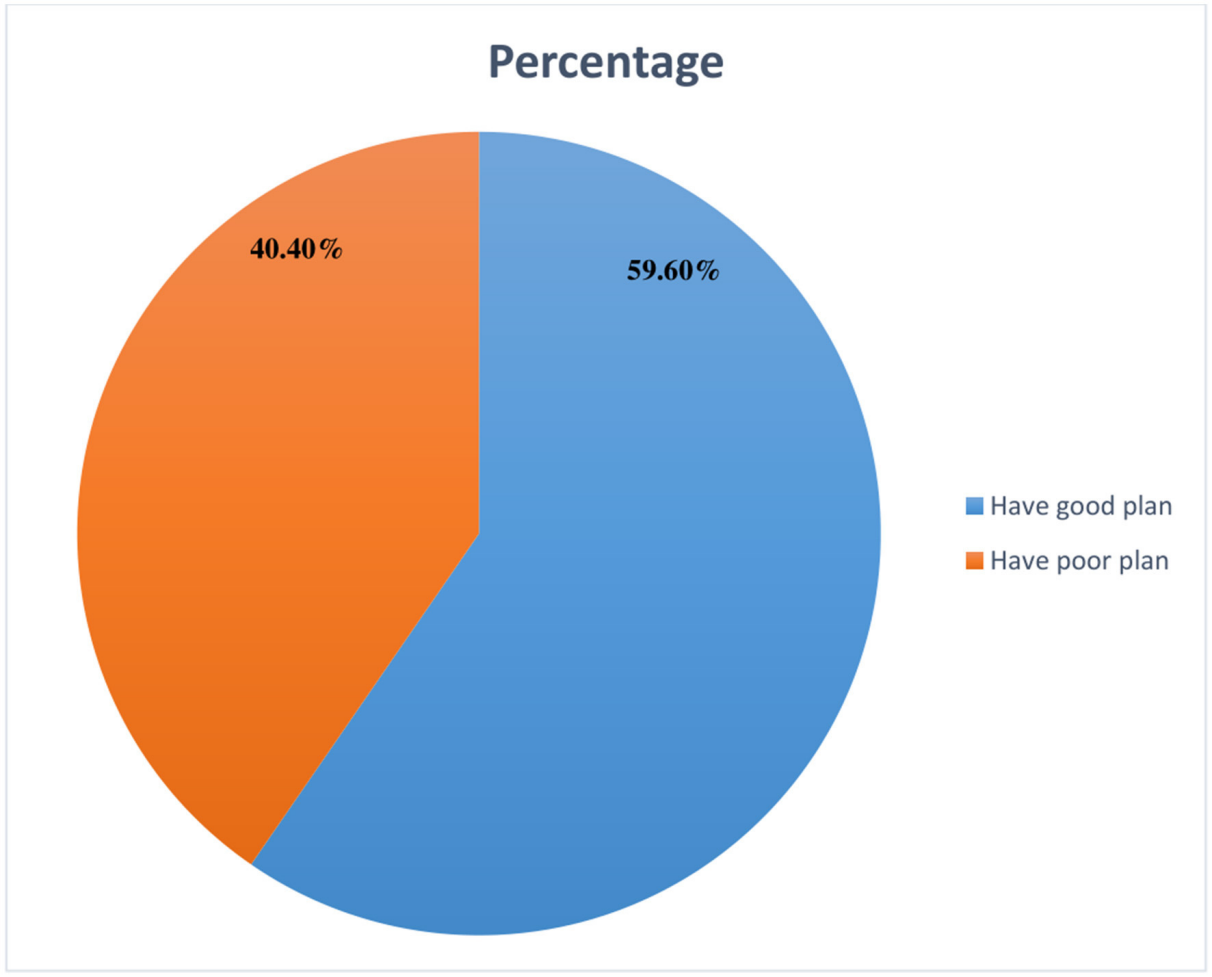
Husband's plan to participate in birth preparedness and complication readiness plan in Haramaya Health and Demographic Surveillance System site, Eastern Ethiopia, 2020.

Moreover, in this study, only 379(60.2%) of husbands had a plan for birth preparedness and complication readiness in the current pregnancy. Of 379 husbands who had birth preparedness and complication readiness plan, around 226 (59.6%) of the husbands had a good participation plan while the remaining 153 (40.4%) of them had a poor participation plan. Thus, the overall proportion of husbands' plans to participate in birth preparedness and complication readiness was 59.6% (95%CI:56–64%) (Figure 3).

### Factors Associated With Husband's Plan to Participate in Birth Preparedness and Complication Readiness

In the bi-variable analysis, predictor variables such as: husband's educational level, husband's occupational status, the importance of ANC, husband's knowledge status on BPCR, knowing danger signs during pregnancy, husband's knowledge of BPCR, husband's knowledge of danger signs during labor and delivery, having a discussion with spouse on the place of delivery, having government support in BPCR and making a plan for PNC follow-up were significantly associated with husband's plan to participate in BPCR in the current pregnancy.

However, in the final model of multivariable logistic regression analysis, predictor variables like husband's knowledge of BPCR, husband's knowledge status of danger signs during labor and delivery, having a discussion with spouse on the place of delivery, and making postpartum plan following delivery were factors remained significantly associated with husband participation in BPCR. Accordingly, husbands who had better knowledge about BPCR were 4.2 times more likely to participate in BPCR than those husbands who had poor knowledge (AOR = 4.18, 95% CI: 2.05, 8.51). Similarly, the likelihood of participating in the BPCR plan was nearly four times higher among husbands who had good knowledge about BPCR than those husbands who had poor knowledge (AOR = 3.99, 95% CI: 2.20, 7.25).

Moreover, the odds of participating in the BPCR plan were 3.19 times higher among husbands who had better knowledge about birth preparedness and complication readiness plan than their counterparts (those husbands who had poor knowledge) (AOR = 3.19, 95% CI; 1.52, 6.71). Likewise, husbands who had good knowledge of danger signs during labor and delivery were nearly three times more likely to participate in BPCR than those who had poor knowledge of birth plan (AOR = 2.84, 95% CI; 1.34, 6.02). Additionally, participants who discussed with their spouse the place of delivery were 6.8 times more likely to participate in BPCR than those who did not discuss the place of delivery with their wives (AOR = 6.84, 95% CI; 4.17, 11.22). Moreover, husbands who had a postpartum plan with their wives were 2.3 more likely to participate in BPCR than those who did not make plans anymore (AOR = 2.3, 95% CI: 1.38, 3.85) (Table 4).

**Table 4:** Bi-variable and multivariable logistic regression analysis of factors associated with husband's participation in BPCR in Haramaya HDSS site, Eastern Ethiopia, 2020.

**Factors**	**Categories**	**Participated in BPCR**		**COR (95% CI)**	**AOR (95% CI)**
		**Yes (%)**	**No (%)**		
Husbands' education level	No formal education	102 (42.5)	138 (57.5)	1	**1**
	Primary (1–8)	129 (66.5)	65 (33.5)	2.68 (1.81, 3.98)	1.59 (0.95, 2.67)
	Secondary (9–12)	70 (76.1)	22(23.9)	4.30 (2.50, 7.41)	1.33 (0.62, 2.87)
	College & above	78 (75.0)	26 (25.0)	4.06 (2.43, 6.77)	0.63 (0.29, 1.38)
Husbands occupation	Farmer	241 (56.0)	189 (44.0)	1	**1**
	Merchant	64 (66.0)	33 (34.0)	1.52 (0.96, 2.41)	0.86 (0.44, 1.67)
	Gov't employee	49 (71.0)	20 (29.0)	1.92 (1.10, 3.34)	0.55 (0.22, 1.37)
	Private employee	25 (73.5)	9 (26.5)	2.18 (0.99, 4.78)	0.80 (0.29, 2.23)
Identify need of ANC during pregnancy	No	6 (18.2)	27 (81.8)	1	**1**
	Yes	373 (62.5)	224 (37.5)	7.49 (3.05, 18.43) ^*^	2.04 (0.63, 6.58)
Have knowledge on danger signs of pregnancy	Poor knowledge	15 (25.9)	43 (74.1)	1	**1**
	Good knowledge	122 (49.2)	126 (50.8)	2.78 (1.47, 5.25)^*^	0.62 (0.25, 1.59)
	Better knowledge	242 (74.7)	82 (25.3)	8.46 (4.47, 16.03)^*^	1.11 (0.40, 3.10)
Have knowledge on BPCR plan	Poor knowledge	23(17.7)	107(82.3)	1	1
	Good knowledge	159 (59.6)	92 (40.4)	6.88 (4.08, 11.60)^*^	3.99 (2.20, 7.25)^**^
	Better knowledge	220 (80.9)	52 (19.1)	19.68 (11.44, 23.86^*^	4.18 (2.05, 8.51)^**^
Have knowledge of danger signs during L & D	Poor knowledge	20 (25.3)	59 (74.7)	1	1
	Good knowledge	109 (54.8)	90 (45.2)	3.57 (2.00, 6.37) ^*^	3.19 (1.52, 6.71) ^**^
	Better knowledge	250 (71.0)	102 (29.0)	7.23 (4.14, 12.62) ^**^	2.84 (1.34, 6.02)^*^
Discussed on a place of delivery with spouse	No	75 (27.8)	195 (72.2)	1	**1**
	Yes	304 (84.4)	56 (15.6)	14.11 (9.56, 20.85) ^*^	6.84 (4.17, 11.22)^**^
Made post natal plan with spouse	No	164 (45.6)	196 (54.4)	1	**1**
	Yes	215 (79.6)	55 (20.4)	4.67 (3.25, 6.71) ^**^	2.30 (1.38, 3.85) ^**^

* *p < 0.01,*

** *p <0.001, L & D, Labor & Delivery; BPCR, Birth Preparedness and Complication Readiness; DS, Danger signs; COR, Crude Odds Ratio, AOR, Adjusted Odds Ratio; CI, Confidence Interval; Bold values are used as reference to determine association in the model*.

## Discussion

This study assessed the husband's plan to participate in birth preparedness and complication readiness and its associated factors in the Haramaya district HDSS site, Eastern Ethiopia. It revealed that the overall prevalence of husbands' plans to participate in BPCR was 59.6%. Thus, approximately two out of every five husbands failed to plan for BPCR. Husband's knowledge of BPCR, husband's knowledge status on danger signs during labor and delivery, having a discussion with spouse on the place of delivery and making postpartum plan were identified as predictors of husband's plan to participate in BPCR.

In this study, around 59.6% of husbands were intended to participate in BPCR during the current pregnancy. Similar findings were reported in a study conducted in the Tigrai region of Northern Ethiopia (60.4 %) (20) and Mekelle Town of Northern Ethiopia (60.9%) (31). The similarities could be due to the fact that the two studies use a similar strategy for safe motherhood and have a similar social structure. However, the current prevalence of husband's plan to participate in BPCR was much higher than previous studies conducted in different settings like Axum, Northern Ethiopia (46.6 %) (18), Bale, Southeast Ethiopia (41.6 %) (32), Nepal (44.36 %) (33), and secondary analysis of DHS data in selected African countries (45.7 %) (5). The possible justification for these disparities might be attributed to differences in sample size, methods of assessment, and the time gaps of the study period. Another possible explanation is that the current study population has better access to information on maternal health care. On contrary, this finding was relatively lower than studies conducted elsewhere such as Gulu district (65.4 %) (12), India (81%) (34), and Nepal (82.6 %) (35). The possible reasons could be due to a variety of socio-demographic factors in the current study setting such as a low level of educational status and socio-economic status of the study participants.

In the final model of multivariable analysis, the husband's knowledge of danger signs during labor and delivery was found to be associated with the husband's plan to participate in BPCR. Thus, those husbands who had a good and better knowledge of danger signs during labor and delivery were 3.19 and 2.84 times more likely to participate in the BPCR plan than those who had poor knowledge, respectively. These results are supported by findings from previous studies conducted in Ethiopia like Burayu (26), Jimma (36), Kofele (37), and other countries such as Nepal (35), and India (38). The possible justification is because knowing about danger signs encourages husbands to seek healthcare service and to participate in BPCR; as improving husbands' awareness and skills could make them involved more in their wives' health status. Moreover, having awareness about potential danger signs of pregnancy may help the husbands to accompany their wives to visit health facilities earlier (39). Moreover, men who are aware of the danger signs of pregnancy and childbirth may become gatekeepers, ensuring that their spouse receives appropriate care in pregnancy-related emergencies (15). Furthermore, when men can recognize danger signs, it makes it easier for women to access health care services, especially in emergencies (9, 12).

Furthermore, in this study, the husband's knowledge status of the birth plan was found to be an independent predictor of BPCR. Accordingly, those husbands with good and better knowledge of BPCRs were 4.18 times and 3.99 times more likely to participate in BPCR than those husbands with poor knowledge respectively. These findings are also supported by studies conducted in Burayu (26) and Nepal (40). This could be because knowing the process of birth preparedness and complication readiness enables husbands to participate in the issues that are beneficial to their spouses. Similarly, the husband's discussion status with their spouse was significantly associated with the husband's plan to participate in BPCR. Thus, those participants who discussed with their wives the place of delivery were 6.84 times more likely to participate in BPCR than those who did not involve in the discussion process. This is in harmony with the findings of the study conducted in Wolaita Sodo, Southern Ethiopia (23). The possible reasons that might be attributed to the household's joint decisions are more powerful because discussing with the husbands could have a positive impact on maternity care services.

Finally, the study pointed out that participants who had made a postpartum plan with their wife were 2.30 times more likely to participate in BPCR than those who had not made a plan. The findings are also in line with a study conducted in Wolaita Sodo, Southern Ethiopia (23), where a higher proportion of husbands who participated in the process of BPCR were observed in those husbands who had no postpartum plan. The possible explanation is that having no postpartum plan was a significant risk factor that endangered or led to the mother's death due to the first two delays in providing care, which is seeking care and reaching a health facility. As a result, preparing for delivery and postpartum care, as well as dealing with unexpected problems, as soon as possible can save the mother's life. Finally, it is very crucial to give more emphasis on BPCR as pregnancy-related complications continue to be a major cause of maternal deaths in Sub-Saharan Africa. Appropriate preparation for birth preparedness and complications readiness by women, male partners, families, and the community has the potential to lower these preventable risk factors. Moreover, policies, programs, and practices could focus on improving male partners' level of knowledge about complications related to pregnancy and childbirth, and the importance of preparing, and planning for childbirth (9, 14).

### Limitations of Study

In this study, due to the nature of the study design, it would be impossible to determine the causal relationship between the variable and the outcome in the analysis. Moreover, as it only involves a participant from rural residences, conclusions for urban could be drawn.

## Conclusion

According to this study, approximately two out of every five husbands failed to plan for BPCR. Husband participation in BPCR was significantly associated with knowledge of BPCR, knowledge of danger signs during labor and delivery, discussion status on the place of delivery, and making a postpartum plan. Therefore, all stakeholders should give more emphasis on male partners' education in terms of birth preparedness and complication readiness, as well as knowledge of danger signs during labor and delivery. It is also very crucial to encourage male partners to discuss a place of delivery and have a postpartum plan in place to reduce potential complications related to labor and delivery. Moreover, we also recommend further community-based longitudinal studies triangulated by qualitative methods to identify and explore predictors of a husband's involvement in BPCR.

## Data Availability Statement

The raw data supporting the conclusions of this article will be made available by the authors, without undue reservation.

## Ethics Statement

The studies involving human participants were reviewed and approved by ethical approval was obtained from Institutional Health Research Ethics Review Committee (IHRERC) of Haramaya University, College of Health and Medical Sciences. The patients/participants provided their written informed consent to participate in this study.

## Author Contributions

All authors made a significant contribution to the work reported, whether that is in the conception, study design, execution, acquisition of data, analysis, and interpretation, or in all these areas, took part in drafting, revising, or critically reviewing the article, gave final approval of the version to be published, have agreed on the journal to which the article has been submitted, and agree to be accountable for all aspects of the work.

## Funding

Haramaya University provided financial support for this study. However, the funding agency had no role in the collection, analysis, and interpretation of the data as well as the writing-up of the manuscript.

## Conflict of Interest

The authors declare that the research was conducted in the absence of any commercial or financial relationships that could be construed as a potential conflict of interest.

## Publisher's Note

All claims expressed in this article are solely those of the authors and do not necessarily represent those of their affiliated organizations, or those of the publisher, the editors and the reviewers. Any product that may be evaluated in this article, or claim that may be made by its manufacturer, is not guaranteed or endorsed by the publisher.
